# Comparison of climbing-specific strength and endurance between lead and boulder climbers

**DOI:** 10.1371/journal.pone.0222529

**Published:** 2019-09-19

**Authors:** Nicolay Stien, Atle Hole Saeterbakken, Espen Hermans, Vegard Albert Vereide, Elias Olsen, Vidar Andersen

**Affiliations:** Faculty of Education, Arts and Sports, Western Norway University of Applied Sciences, Sogndal, Norway; University of Brasilia, BRAZIL

## Abstract

Albeit differences in climbing-specific strength of the forearms have been demonstrated between lead and boulder climbers, little is known about the potential differences in force and power output of the upper body pulling-apparatus between disciplines. The aim of this study was to compare the climbing-specific upper-body strength and finger flexor endurance between lead and boulder climbers, as well as to examine the relative utilization of force when testing on a ledge hold compared to a jug hold. Sixteen boulder climbers (red-point climbing grade 17.9 ± 3.3) and fifteen lead climbers (red-point climbing grade 20.5 ± 3.5) performing on an advanced level volunteered for the study. Peak force, average force and rate of force development (RFD) were measured during an isometric pull-up, average velocity in dynamic pull-up, and finger flexor endurance in an intermittent test to fatigue. The isometric pull-up was performed on a ledge hold (high finger strength requirements) and on a jug hold (very low finger strength requirements). Boulder climbers demonstrated a higher maximal and explosive strength in all strength and power measurements (26.2–52.9%, *ES* = 0.90–1.12, *p =* 0.006–0.023), whereas the finger flexor endurance test showed no significant difference between the groups (*p =* 0.088). Both groups were able to utilize 57–69% of peak force, average force and RFD in the ledge condition compared to the jug condition, but the relative utilization was not different between the groups (*p =* 0.290–0.996). In conclusion, boulder climbers were stronger and more explosive compared to lead climbers, whereas no differences in finger flexor endurance were observed. Performing climbing-specific tests on a smaller hold appears to limit the force and power output equally between the two groups.

## Introduction

Sport climbing and bouldering have greatly increased in the last decades [1]. Competitive climbing consist of three disciplines; lead climbing, bouldering and speed climbing. Of the three, lead climbing and bouldering currently are the two most practiced disciplines [2]. Indoor bouldering routes typically consist of less than eight-to-ten climbing moves and is performed without ropes on a less than five-meter high wall [2, 3]. Lead climbing consists of multiple climbing moves and is performed on higher walls (>10m) [4]. Still, only a few studies have compared the physical characteristics of climbers specializing in the two disciplines [3, 5–7].

Factors such as hold type (size and shape) and the gradient of the wall determine the difficulty of boulder and lead climbing routes [8–11]. In order to apply the force generated from the back, shoulder and arm muscles (prime movers) to the holds during moves, sufficient finger strength is required. Therefore, it is generally accepted that finger flexor strength is a crucial factor for performance in climbing and climbing-specific tests [3, 5, 12–21]. For example, Vigouroux et al. [20] demonstrated that smaller climbing holds have a negative impact on force and power output in addition to number of pull-ups to failure among elite climbers. To the authors’ knowledge, however, no previous studies have examined the percentage of isometric force generated by the prime movers that can be utilized on a ledge hold (high level of finger strength requirement) compared to a jug hold (very low level of finger strength requirement). If there is room for improved utilization of force through increased finger strength, it is possible that an augmented climbing performance can be achieved without increasing the strength of the prime movers.

Whereas the average contact times in climbing competitions are 8–10 seconds [2, 4], the typical ledge dead-hang time for an elite climber is over 60 seconds. Therefore, tests attempting to mimic the typical contraction/relaxation ratios used in sport climbing have been developed. Specifically, 40% of max force in a 10:3 ratio [5, 17, 18, 22], 60% of max force in an 8:3 ratio [19, 23] and 80% of max force in a 5:5 ratio [24] have been used in previous studies. These intermittent forearm muscle endurance tests have demonstrated greater endurance among climbers compared to non-climbers, but no difference between climbing disciplines [5, 17–19, 22–24].

In addition to forearm muscle endurance and maximal strength, the ability to exert force quickly has been suggested as a crucial component for climbing performance [9, 25, 26]. Especially for performing long moves and quickly having to grip a hold [26]. One frequently used parameter to examine explosive strength characteristics is rate of force development (RFD) [27]. Still, only one study has examined RFD as a measure for detecting differences between climbers of different disciplines [3]. The authors demonstrated that boulder climbers were able to develop finger flexor force at a higher rate than lead climbers and that finger flexor peak RFD was a crucial factor for discriminating between the two disciplines [3]. Contrastingly, the mean RFD value has been suggested as a more accurate measurement than peak values for assessing climbing ability due it being less sensitive to variability [26]. Still, little is known about the rapid force production characteristics of the prime movers in a climbing-specific test among lead and boulder climbers.

Previous studies comparing lead and boulder climbers have been limited by few test parameters focusing mainly on the finger flexors, and only one study has examined the effect of hold size when performing pull-ups [20]. Thus, a negligible knowledge exists about the upper-body strength characteristics of lead and boulder climbers and the impact of hold type on the utilization of the force generated by the prime movers during climbing-specific tasks. The aims of the present study were, therefore, 1) to examine maximal and explosive strength in dynamic and isometric pull-up, 2) to identify the utilization rate of force using a ledge hold compared to a jug hold, and 3) to compare forearm muscle endurance between lead and boulder climbers. On the basis of previous research [3, 5] and the specificity of the two disciplines, boulder climbers were hypothesized to demonstrate greater maximal force, RFD and pull-up velocity, while lead climbers were expected to demonstrate greater climbing-specific forearm muscle endurance. Both groups were also expected to demonstrate reduced force output and RFD in the isometric pull-up using the ledge hold compared to the jug hold.

## Materials and methods

### Study design

To determine the possible differences in forearm muscle endurance and climbing-specific strength characteristics (maximal and explosive) between lead and boulder climbers, a cross-sectional study was conducted with group as the independent variable. The climbers were tested for maximal isometric pull-up strength (average rate of force development (RFD_avg_), Peak force (F_peak_) and average force (F_avg_)), explosive dynamic pull-up strength (average velocity (V_avg_)), and finger flexor endurance (intermittent test to fatigue) during one laboratory session. All subjects performed the tests in a standardized order: 1) isometric pull-up on a ledge, 2) isometric pull-up on a jug, 3) dynamic pull-up on a ledge, and 4) intermittent test. Three to five minutes of rest was allowed between each trial and test condition. All tests were performed bilaterally. The subjects were instructed to refrain from climbing and climbing-related training for 48 hours before testing.

### Subjects

Thirty-one recreational climbers (28 males and 3 females) volunteered for the study and were allocated to the boulder climbers (*n* = 16) or lead climbers (*n* = 15) groups, based on their self-reported main practiced discipline. For details of anthropometric data, self-reported climbing ability and number of weekly climbing sessions, see Table 1. Climbing ability, experience and number of weekly sessions were not different between the groups (*p =* 0.056–0.401). The subjects reported their climbing ability using the French grade system (1-9a/b/c) and the grades were converted into the numeric reporting scale (1–32) proposed by the International Rock Climbing Research Association (IRCRA) [28]. The minimal self-reported accomplished climbing grade (red-point) to be included in the study had to be no less than 7a (IRCRA 17) for men and 6b (IRCRA 13) for women. All subjects were informed about the study orally and in writing and signed an informed consent form prior to collection of data. The consent form and the testing procedures were confirmed with the Regional Committees for Medical Health and Research Ethics in Norway (2018/1345 REK Sør-øst D), were in accordance with the ethical guidelines of Western Norway University of Applied Sciences and conformed to the standards of treatment of human participants in research, outlined in the 5^th^ Declaration of Helsinki.

**Table 1:** Anthropometric data, number of weekly climbing sessions, climbing experience and self-reported climbing ability (IRCRA scale). Data are given as mean (± SD).

	Boulder Climbers 15 male, 1 female	Lead Climbers 13 male, 2 female
**Age (years)**	25.31 (3.44)	28.60 (6.72)
**Height (cm)**	178.52 (7.90)	175.77 (7.00)
**Body mass (kg)**	72.29 (7.80)	68.52 (8.53)
**Fat (%)**	11.68 (4.20)	12.17 (4.13)
**Fat-free mass (%)**	83.93 (3.99)	83.45 (3.93)
**Climbing experience (years)**	5.91 (4.58)	9.00 (4.58)
**Weekly climbing sessions**	3.88 (1.61)	3.43 (1.24)
**Red-point (IRCRA)**	17.85 (3.4)	20.47 (3.54)

### Procedures

#### Body composition and anthropometric data

Body composition (relative fat mass and fat-free mass) and body mass were measured using a bioelectrical impedance scale (Tanita MC 780MA S, Tokyo, Japan) with the subjects wearing light clothing and no shoes. Body height was measured using a wall mounted measuring tape. Body composition and anthropometric data were not different between the two groups (*p =* 0.211–0.736; see Table 1).

#### Isometric pull-up

**Fig 1:**
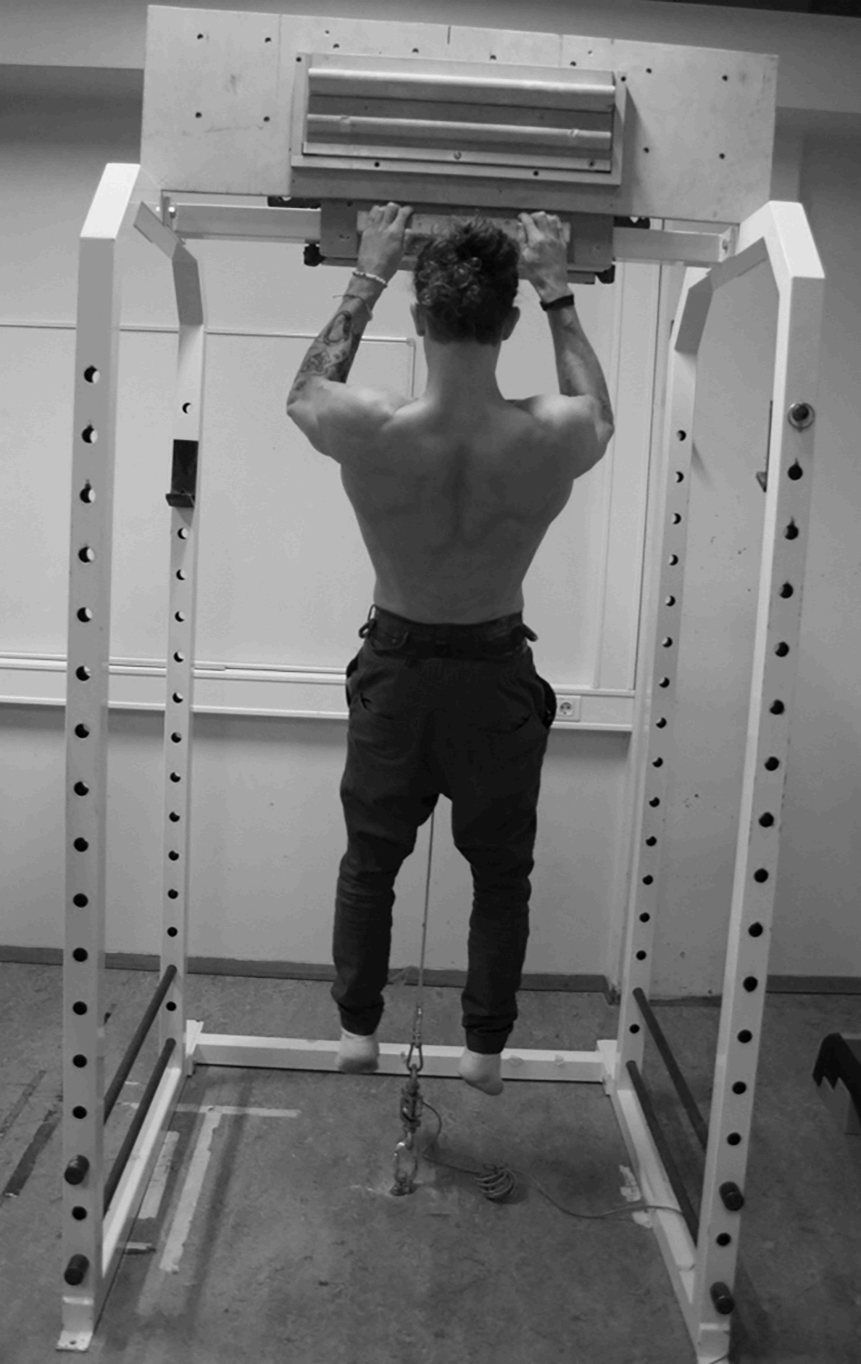
Position with 90 degrees elbow flexion for the isometric pull-up.

**Fig 2:**
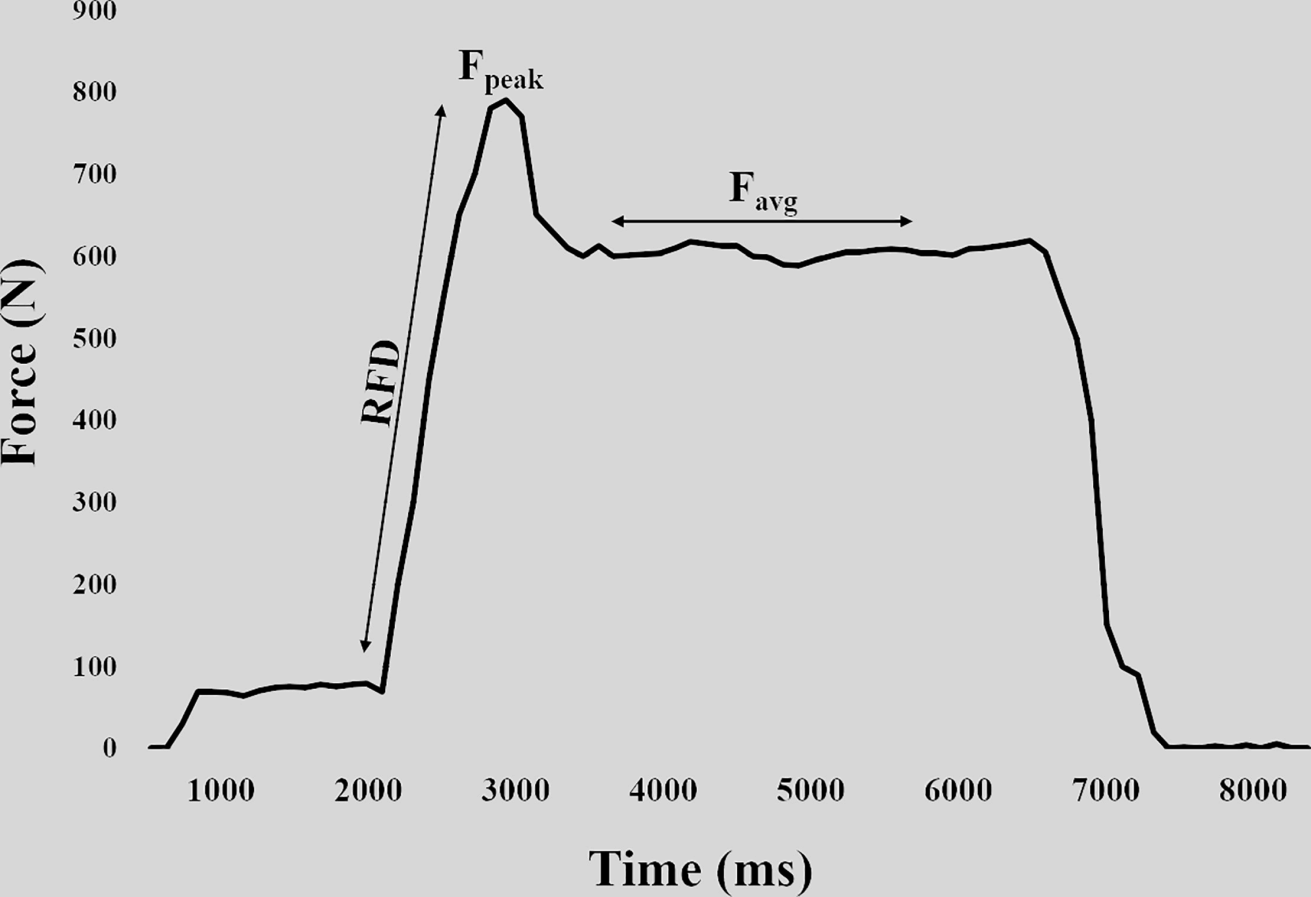
Schematic force curve produced in the isometric pull-up. Markers indicate rate of force development (RFD_avg_), peak force (F_peak_) and average force (F_avg_).

Before measuring strength performance, the subjects performed a 15-minute warm-up consisting of bouldering and traversing on self-selected routes. The subjects were instructed to maintain a light-to-moderate intensity in the warm-up to avoid fatigue. The F_avg_, F_peak_ and RFD_avg_ measurements were collected from the same attempt, performing a five seconds maximal, isometric pull-up with elbows flexed at 90 degrees and an open crimp grip (Fig 1). To measure force output, subjects wore a climbing harness anchored to the floor via a static rope and a force sensor (Ergotest Innovation A/S, Porsgrunn, Norway) to remain in an isometric position. The length of the rope between the climbing harness and force sensor was adjusted to maintain a correct elbow angle (measured with goniometer). The subjects pulled themselves up to a 90 degrees angle (i.e. where the static rope became taut) in the elbow joint and maintained the position for approximately one second before being verbally encouraged to perform a maximal isometric pull-up and maintain maximal force for five seconds. This test produces a clear F_peak_ early in the contraction (Fig 2). Elastic components within the muscles and a small shuttle caused by alterations of the shoulder and elbow joints when applying maximal force likely contribute to the prominent F_peak_.

**Fig 3:**
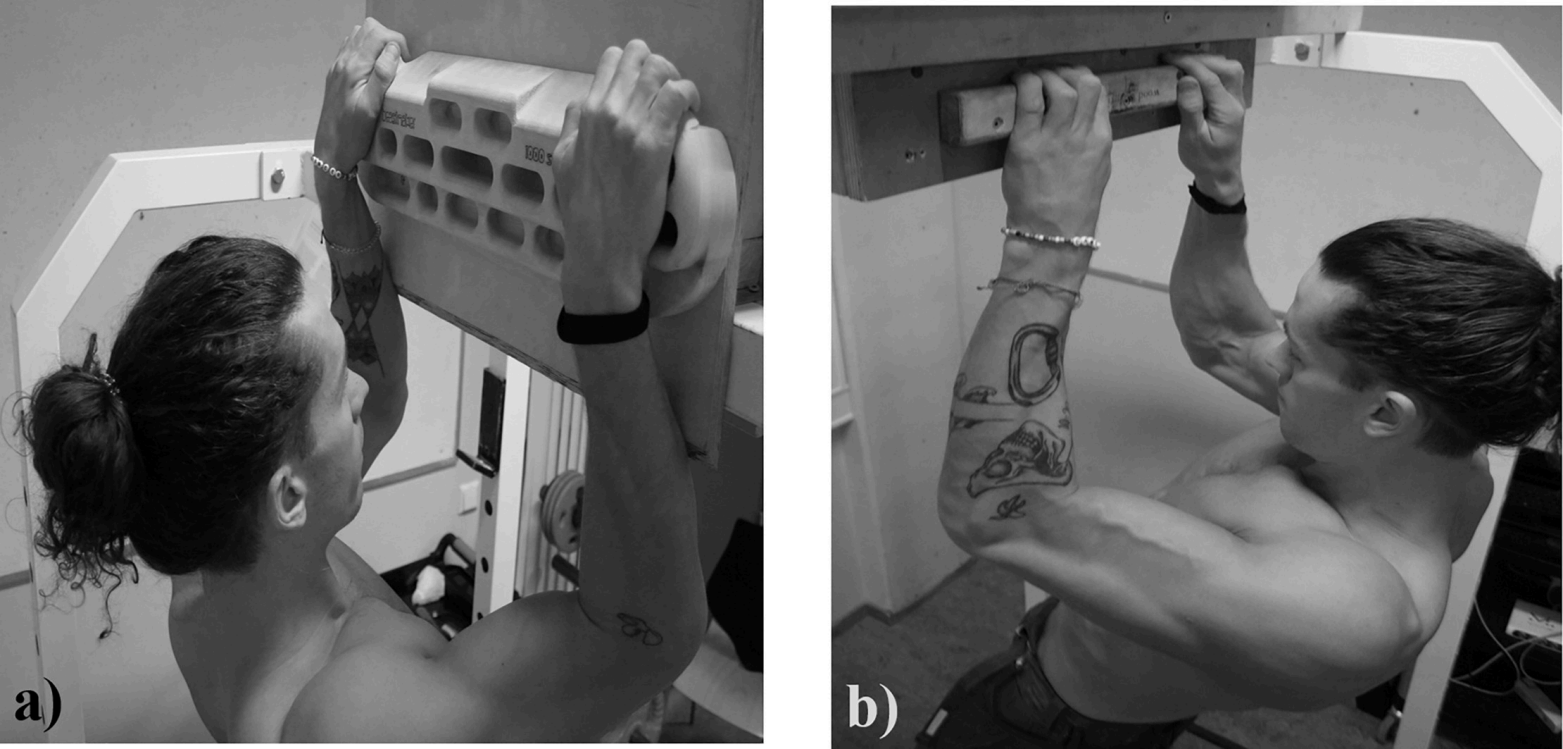
**The isometric pull-up using the jug (a) and ledge (b)**.

The testing procedures were conducted using two different conditions; 1) On a wooden jug grip (Fig 3A) (Beastmaker 1000 series, Beastmaker Limited, Leicester, United Kingdom), and 2) on a 43 cm wide and 23 mm deep wooden ledge with rounded edges (Fig 3B) (Metolius Climbing, Bend, Oregon, USA). The ledge was regularly brushed to provide equal friction conditions for all subjects. Three attempts were given in each condition, with one minute rest between each attempt and three minutes rest between conditions [29]. The results from the best attempt for each condition was used in the analyses. The F_avg_, F_peak_ and RFD_avg_ were recorded by the force sensor at 200Hz and analyzed with the MuscleLab software (v. 10.4.37.4073, Ergotest Innovation A/S, Porsgrunn, Norway). RFD_avg_ was calculated as the mean increase in force from the onset of force generation after pulling themselves up to the 90 degrees elbow angle and to the F_peak_ (Fig 2). The onset of force was determined visually, which has been proposed as more sensitive and accurate than automated detection [30]. The F_peak_ was registered from the highest force output on the curve and F_avg_ was calculated as the mean force over a two seconds period, excluding the peak (Fig 2). The recorded force values including the gravitational force of the body (body mass × 9.807) were used in the analyses. The relative utilization of force on the ledge relative to the jug was calculated as follows; ((ledge results / jug results) × 100).

#### Dynamic pull-up

The V_avg_ was measured during a dynamic pull-up performed on the same ledge used in the isometric pull-up test (Metolius Climbing, Bend, Oregon, USA). The subjects performed one concentric pull-up as fast as possible from a dead-hang position (elbows fully extended) until the eyes were above the hands. Kipping with the legs was not allowed. A linear encoder (ET-Enc-02, Ergotest Innovation A/S, Porsgrunn, Norway) with a resolution of 0.075 mm and counting the pulses with a 10-millisecond interval was attached to a climbing harness and recorded the displacement of the body and the movement velocity performing the pull-up. The results were instantaneously analyzed using the MuscleLab software (v. 8.13, Ergotest Innovation A/S, Porsgrunn, Norway). One-minute rest was given between each attempt and the results from the attempt with the highest values were used in the analyses.

#### Intermittent forearm muscle endurance test

**Fig 4:**
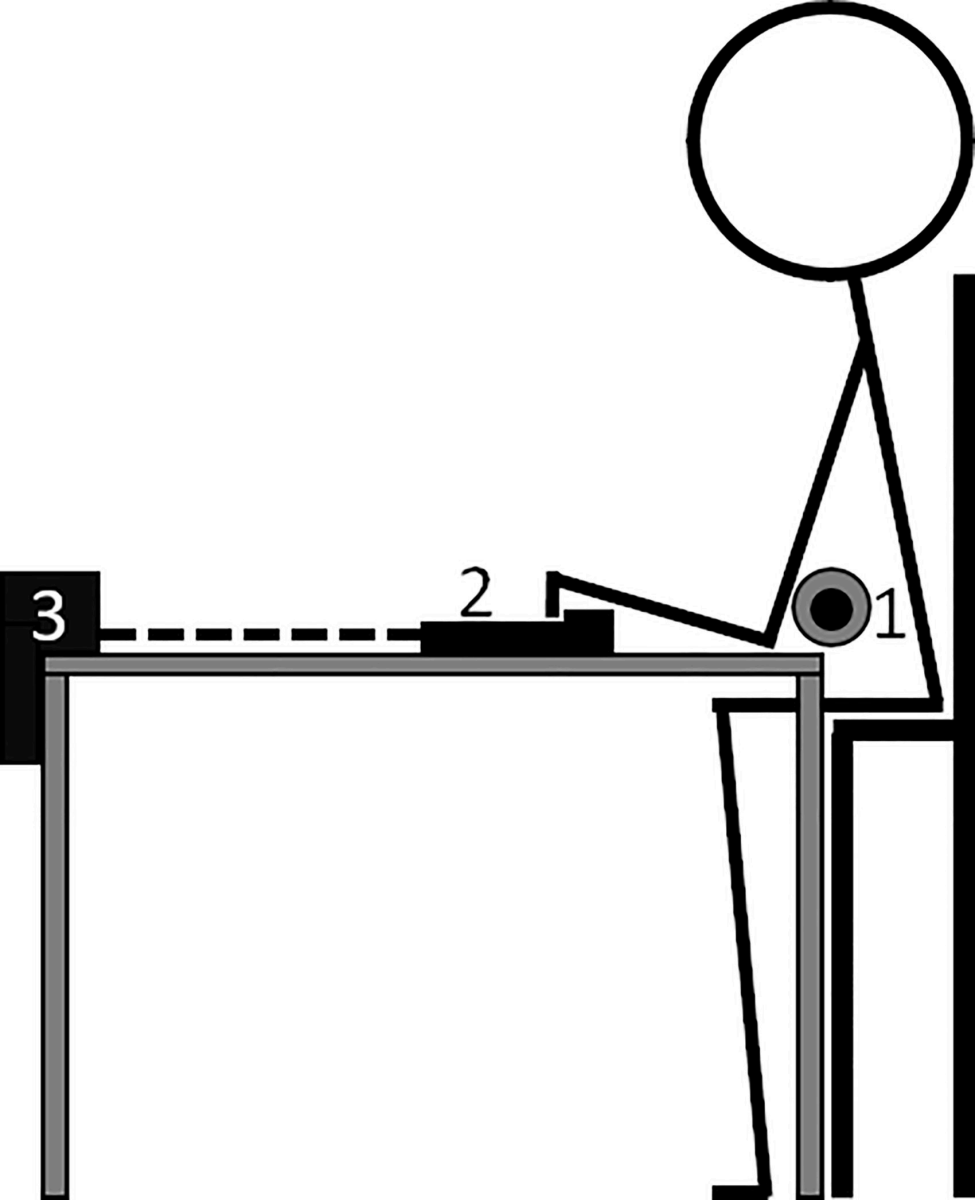
Set-up for the intermittent forearm muscle endurance test. The figure depicts the subject in the seated position with 1) the padded barbell limiting involvement of the back muscles, 2) the wooden ledge and 3) the force cell.

The intermittent forearm muscle endurance test was conducted in a seated position with the shoulders fully adducted, a 90 degrees flexion in the elbow and an open crimp grip (Fig 4). A padded barbell was placed in front of the subjects’ chest and behind the distal part of the upper arms to prevent any movement or involvement of the shoulders or back muscles. A 46 cm wide and 23 mm deep custom-built wooden ledge was attached to a force cell (Ergotest Innovation A/S, Porsgrunn, Norway) that measured the applied force of the finger flexors to the ledge. Before starting the intermittent test, the subjects’ maximal isometric finger flexion force was determined in the same position. The test consisted of seven seconds contraction at 60% of maximal isometric force intermittently with three seconds rest (7:3 ratio) to fatigue. This contraction/relaxation ratio is similar to that observed in climbing competitions [2, 4]. Between each bout of work, the subjects could rest, but not let go of the ledge or change their grip technique. A phone application (Beastmaker Training App v. 2.0.1, Beastmaker Limited, Leicester, United Kingdom) and a computer screen mirroring the MuscleLab software (v. 10.4.37.4073, Ergotest Innovation A/S, Porsgrunn, Norway) were placed in the subjects’ field of vision to give visual information about work/rest periods and real-time feedback of the generated force. The researchers also gave verbal start-and-stop instructions. Watching the computer screen, the subjects were able to adjust their applied force to the hold continuously. If the force dropped below their individual threshold value for more than a second, the test was stopped [23]. The total effective work time was used in future analyses.

### Statistical analyses

Except from V_avg_ in the dynamic pull-up (Shapiro-Wilk test; *p =* 0.001), no other variables revealed deviations from normality (*p =* 0.060–0.946). SPSS statistical software (Version 25.0, SPSS Inc., Chicago, IL, USA) was used for the analyses. Differences between the groups were identified using an independent student’s *t*-test for the parametric variables and using a Mann-Withney U Test for the non-parametric variable (i.e. V_avg_). For statistical significance, the alpha level was set at 0.05. The data is presented as mean (± SD) and Cohen’s d effect size (*ES*). An *ES* of < 0.2 was considered trivial, 0.2 small, 0.5 medium, and > 0.8 large [31].

## Results

**Fig 5:**
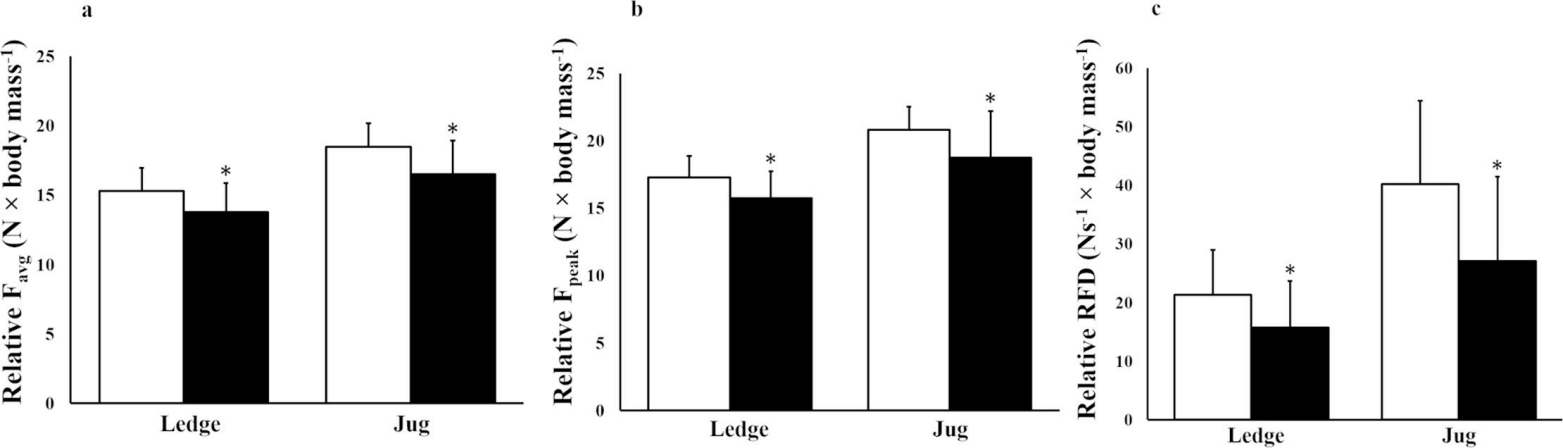
**Results relative to body mass from average force (F**_**avg**_**) (a), peak force (F**_**peak**_**) (b) and rate of force development (RFD**_**avg**_**) (c) between the groups for the isometric ledge and jug conditions.** White bars represent boulder climbers and black bars represent lead climbers. Error bars represent standard deviations. (* *p* < 0.05; ** *p* < 0.01).

In the isometric tests, boulder climbers demonstrated 28.7–52.9% higher F_peak_, F_avg_ and RFD_avg_ than lead climbers using the 23 mm ledge and the jug (*p =* 0.013–0.015; see Table 2). Boulder climbers also demonstrated significantly higher F_peak_, F_avg_ and RFD_avg_ than lead climbers (23.1–48.4%, *ES* = 0.78–0.97, *p =* 0.016–0.044) when the data was analyzed relative to the body mass (Fig 5A, Fig 5B, Fig 5C and S1 File).

**Table 2:** Absolute values from the dynamic and isometric pull-up and the forearm endurance test.

		Boulder Climbers		Lead Climbers			
		Mean	SD	Mean	SD	*P*	ES
**Isometric ledge**							
	F_peak_ (N)	1249	175	1079*	187	0.014	1.06
	F_avg_ (N)	1106	175	946*	172	0.015	0.99
	RFD_avg_ (Ns^-1^)	1537	548	1057*	485	0.013	0.96
**Isometric jug**							
	F_peak_ (N)	1503†	185	1287*†	308	0.025	0.90
	F_avg_ (N)	1334†	181	1131*†	228	0.011	1.12
	RFD_avg_ (Ns^-1^)	2869†	939	1876*†	1050	0.006	1.03
**Utilization rate**							
	F_peak_ (%)	69	14	69	12	0.996	0.00
	F_avg_ (%)	64	16	58	16	0.290	0.39
	RFD_avg_ (%)	57	20	64	27	0.476	0.61
**Dynamic pull-up**							
	V_avg_ (ms^-1^)	0.96	0.26	0.76*	0.15	0.014	0.97
**Intermittent test**							
	Time (s)	83	18	107	40	0.088	0.78

The results are presented as mean (± SD) with Cohen’s d effect size (ES) and *P* value for the difference between groups. F_peak_ = peak force, F_avg_ = average force output, RFD_avg_ = rate of force development from the onset of force to the peak force output, utilization rate = ledge performance relative to jug performance, V_avg_ = average velocity, Time = total work time to fatigue.

* = Significantly lower than boulder climbers (*P* < 0.05).

† = Significantly different from ledge condition (*P* < 0.01).

Both groups demonstrated lower F_peak_, F_avg_ and RFD_avg_ in the ledge condition compared to the jug condition (*ES* = 1.79–2.02, *p* < 0.005). However, the utilization of force in the ledge condition relative to the jug condition (57–69%) was not different between the groups (*p =* 0.290–0.996).

In the dynamic pull-up test, boulder climbers achieved a 26.2% higher V_avg_ than lead climbers (*p =* 0.014; see Table 2, S1 File).

In the intermittent forearm muscle endurance test, the time to fatigue demonstrated no difference between lead and boulder climbers (*p =* 0.088; see Table 2, S1 File)

## Discussion

In accordance with the hypothesis, the boulder climbers exhibited greater maximal (F_peak_ and F_avg_) and explosive strength (V_avg_ and RFD_avg_) than the lead climbers, whereas the analyses showed no difference in forearm muscle endurance between the groups.

Greater maximal isometric strength (F_peak_ and F_avg_) for the boulder climbers compared to lead climbers was not surprising. Importantly, lead climbing is typically performed more static with slow and controlled movements over a longer period of time than bouldering [2]. Contrastingly, bouldering contains steep, but short routes and a higher frequency of moves performed with maximal effort [2, 3, 8, 32]. Thus, especially regarding intensity and repetitions per set (moves per attempt), the physiological demands of bouldering are more similar to the recommendations for maximal strength training [33] and is likely a more appropriate training stimulus than lead climbing for improving force output [7, 34]. This was demonstrated by a 29–45% greater absolute and relative strength (force / body mass) for the boulder climbers compared to the lead climbers. Interestingly, both groups demonstrated a similar utilization rate in the ledge condition relative to the jug condition (57–69% of jug results), suggesting that despite boulder climbers being stronger in the prime movers, both groups are equally limited by finger strength when testing on a smaller hold. These results support previous findings that the hold size and shape is a crucial factor for performance in climbing-specific tests [20].

Frequent explosive moves on steep walls require boulder climbers to apply force rapidly to perform dynamic moves and ensure the subsequent stabilization of the body [2, 35]. Additionally, the available time to generate force during bouldering (contact time) will often be shorter than the time it takes to reach maximal force [25]. Hence, being able to generate as much force as possible in a short time is crucial for performance in bouldering. Therefore, RFD has been proposed as an important discriminatory factor between lead and boulder climbers [2, 3, 9]. The current observations support this claim, with RFD_avg_ demonstrating the largest difference between the two groups (38–53%). Likewise, the dynamic measure of explosive strength (V_avg_) was 26% higher for the boulder climbers compared to the lead climbers. The marked differences for explosive strength parameters between the groups likely reflect the specific requirements of the two disciplines [34]. As bouldering offers a higher frequency of explosive and gymnastic moves than lead climbing [2, 36], this sub-discipline of climbing is likely better suited for improving RFD through mechanisms such as increased firing rate of motor units, changes in muscle fiber composition or muscle-tendon stiffness [27, 37]. Repeated exposure to these stimuli have likely resulted in chronic adaptations that separate boulder climbers from lead climbers. However, and as pointed out by a previous study [3], one cannot rule out the possibility that climbers have chosen their discipline based on their predisposed abilities. The present study supports several previous findings [3, 5–7], suggesting boulder climbers are stronger and more explosive compared to lead climbers. However, further research is needed to determine whether this is a result of genetic predisposition or distinct adaptations to performing lead or boulder climbing.

While a boulder route ascend typically lasts around 30 seconds, leading a route may take anywhere between 2–7 minutes [2, 9]. Therefore, we expected forearm muscle endurance to distinguish between climbing disciplines. However, the present findings showed no difference in forearm muscle endurance between lead and boulder climbers. In the intermittent test, 60% of the maximal finger flexor force had to be generated in the seven seconds work period. This resulted in a mean work time of 107 seconds vs 83 seconds for lead and boulder climbers, respectively. It is possible that the short work time was not sufficient to detect differences in forearm muscle endurance between the climbing disciplines. For example, Fryer et al. [22] used 40% of maximal force in a 10:3 contraction/rest ratio, which resulted in a work time of 264–332 seconds in subjects with a similar climbing performance level as the present study population. An endurance test with longer duration or lower force threshold could, therefore, be more useful for distinguishing between climbers of the two disciplines [19]. Contrastingly, Vigouroux et al. [24] used a 5:5 ratio with 80% of maximal force as the threshold and demonstrated a higher forearm muscle endurance among elite climbers compared to non-climbers (180 vs. 90 seconds work time). Owing to the lower performance level among the current study population, however, a higher force threshold with a 7:3 ratio might have been too heavy for the subjects and reduced in a much shorter work time. Furthermore, by increasing or decreasing the threshold and thereby changing the total work time, the test would likely favor either boulder climbers or lead climbers, respectively. Lastly, albeit the subjects performed on an advanced level, the current study population consisted of recreational climbers. It is possible that more distinct differences could be observed in elite climbers that are more specialized in their respective disciplines.

Some potential limitations of this study should be outlined. Only recreational climbers were recruited and, therefore, the findings cannot necessarily be generalized to other populations of climbers. Furthermore, the subjects were not asked to report their bouldering performance level. By examining potential differences in lead and boulder climbing ability, it could have been possible to determine the specific importance of different physical attributes for the two disciplines. Lastly, the slope of the force curve displayed an elevated F_peak_ compared to F_avg_. The prominent F_peak_ likely occurred owing to elastic components within the muscle and displacement of the joints when applying maximal force from a hanging position. Although this force curve may be perceived as unusual, the test set-up closely mimics the mechanics of climbing where forceful moves are performed from a hanging position with pre-activation of the muscles.

In conclusion, the current findings demonstrated greater maximal and explosive strength in the boulder climbers compared to the lead climbers, whereas no differences were noted for forearm muscle endurance. As RFD revealed the most marked difference between the two disciplines, this measure should be considered in future studies assessing muscular characteristics among rock climbers. The results likely reflect the specific adaptations to the physical demands of the two disciplines with regard to ascent duration, number of moves, distance between holds and steepness of the route.

### Practical applications

Differences in isometric and dynamic strength characteristics were observed in a study population performing on an advanced level (~19 IRCRA scale). A rather novel finding in this study was that maximal and explosive strength in both dynamic and isometric pull-up are higher among boulder climbers compared to lead climbers. On the basis of the current findings, bouldering might be a more appropriate training stimulus for maximal and explosive strength compared to lead climbing. The specific adaptions and strength characteristics of the two climbing disciplines need to be recognized and emphasized among trainers. As both groups demonstrated a relative force utilization of 57–69% in the ledge condition relative to the jug condition, finger flexor strength training can likely benefit climbers of both disciplines. Resistance training focusing on maximal and explosive strength of both the finger flexors and the prime movers can likely benefit both groups, but might prove more beneficial for boulder climbers than lead climbers. Lead climbers might benefit more from focusing their training on other properties, such as forearm muscle endurance. Future research should further examine the maximal and explosive strength of the prime movers and examine the relative utilization of the force generated from the prime movers on different holds among climbers of different disciplines on an elite level.

## Supporting information

S1 File (XLSX)
